# Distinct Acute Zones for Visual Stimuli in Different Visual Tasks in *Drosophila*

**DOI:** 10.1371/journal.pone.0061313

**Published:** 2013-04-09

**Authors:** Xing Yang, Aike Guo

**Affiliations:** 1 Institute of Neuroscience and State Key Laboratory of Neuroscience, Shanghai Institutes for Biological Sciences, Chinese Academy of Sciences, Shanghai, China; 2 Graduate School of Chinese Academy of Sciences, Beijing, China; 3 State Key Laboratory of Brain and Cognitive Science, Institute of Biophysics, Chinese Academy of Sciences, Beijing, China; Universitaet Regensburg, Germany

## Abstract

The fruit fly *Drosophila melanogaster* has a sophisticated visual system and exhibits complex visual behaviors. Visual responses, vision processing and higher cognitive processes in *Drosophila* have been studied extensively. However, little is known about whether the retinal location of visual stimuli can affect fruit fly performance in various visual tasks. We tested the response of wild-type Berlin flies to visual stimuli at several vertical locations. Three paradigms were used in our study: visual operant conditioning, visual object fixation and optomotor response. We observed an acute zone for visual feature memorization in the upper visual field when visual patterns were presented with a black background. However, when a white background was used, the acute zone was in the lower visual field. Similar to visual feature memorization, the best locations for visual object fixation and optomotor response to a single moving stripe were in the lower visual field with a white background and the upper visual field with a black background. The preferred location for the optomotor response to moving gratings was around the equator of the visual field. Our results suggest that different visual processing pathways are involved in different visual tasks and that there is a certain degree of overlap between the pathways for visual feature memorization, visual object fixation and optomotor response.

## Introduction

The fruit fly *Drosophila melanogaster* has a sophisticated visual system and exhibits complex visual behaviors. Several types of visual behaviors have been studied, including classic optomotor responses [1]–[4], saccades [5]–[7] and landing [6], [8] as well as sophisticated behaviors such as feature extraction [9] and decision making [10]. However, the effect of the location of visual stimuli on fruit fly behavior has not been well studied.

The primate eye contains a fovea, and the primate visual system is more sensitive to the details of objects: shape, color and texture in the inner visual field and motions in the peripheral visual field are more easily detected [11]–[13]. The eyes of male hoverfly *Eristalis tenax* have a region of large facets that form a bright zone with increased light capturing ability [14]. Some fast-flying insects such as blowflies and dragonflies have a region with a high density of ommatidia in their eyes. This region, which is referred to as the acute zone, is useful for hunting prey or for pursuing potential mates in flight [15], [16]. Although the *Drosophila* eye does not contain obvious large facets, the ommatidia are not homogeneously distributed and are densest in the frontal area around the equator of the eyes, while the left and right eyes share a small receptive field in the frontal area [17]. The *Drosophila* compound eye is composed of nearly 800 ommatidia; those ommatidia are not functionally identical. As different opsins are expressed in R7/R8, the ommatidia have at least 3 subtypes that exhibit different color preferences. Although the 2 subtypes that exhibit a blue or green preference are randomly distributed around the eye, the subtype that only has an ultraviolet preference and that is specialized to polarized skylight is restricted to the dorsal rim area [18]. However, ommatidia in the ventral eye were recently reported to mediate polarotactic responses in *Drosophila* [19].

At the behavioral level, the optomotor responses of tethered flies to black stripes change with the azimuth angle of the stripes. Visual cues can direct the attention of a fly to a certain area. In the lower visual field, the attention effect can be observed even when the cues precede the test stimulus by several seconds and is spatially separated from the test stimulus [20]. During closed-loop conditioning in a flight simulator, flies spontaneously orient themselves to a black stripe by modulating yaw torques to keep the black stripe in the frontal area of their visual field, and it has been reported that only the stripes below the equator of their eyes could be fixed adequately by house flies [21]. In this study, we selected 3 paradigms: visual operant conditioning [22], [23], optomotor response and object fixation behavior [17], [24], [25]. These paradigms are often used to study the vision-processing mechanisms of flies [26] and even circuit mechanism in advanced cognitive behavior [9], [10], [24], [27], [28]. However, studies of the location effects of visual stimuli have been limited. We studied the performance of wild-type Berlin (WTB) flies in response to visual stimuli at different vertical locations in a flight simulator, and our results indicated that there were distinct sensitive regions in the visual field for these paradigms. By analogy with the high acuity region in several dipterans [15], these sensitive regions could also be termed acute zones. The acute zone for visual feature memorization was in the upper visual field with a black background and in the lower visual field with a white background. The acute zones for object fixation and optomotor response while following a single moving stripe were similar to that for visual feature memorization, whereas the preferred location for optomotor response while following moving gratings was located at approximately the equator of the visual field. These results suggest that there is a certain degree of overlap between the pathways for visual feature memorization, visual object fixation and optomotor response. Despite the overlap, our analysis of the torque spikes suggests that the torque spike modulation is only required for memory acquisition of visual feature.

## Materials and Methods

### Fly stocks

WTB flies were raised on standard medium at 25°C and 60% relative humidity in a 12 h/12 h light/dark cycle [29]. On the day before the experiment, female flies (aged 3–5 days) were briefly immobilized with cold anesthesia. Their heads were then glued (Loctite UV glass glue) to their thoraxes, and small, triangular hooks (0.05 mm in diameter) were glued to the frontal dorsal part of the thorax. Flies were then kept individually in small chambers and fed with a sucrose water solution until the experiment. Before the flies were fixed to a torque meter, they were fastened to an aluminum clamp, and the clamp was fastened to the electrode manipulator in an MF-830 microforge with a scale eyepiece. The handle of the clamp was in vertical orientation. The elevation angles of their heads were then adjusted to zero with forceps in the MF-830 microforge (Fig. S1). The flies with the clamp were then affixed to a torque meter and the handle of the clamp was still in vertical orientation.

### Operant conditioning paradigm

**Figure 1:**
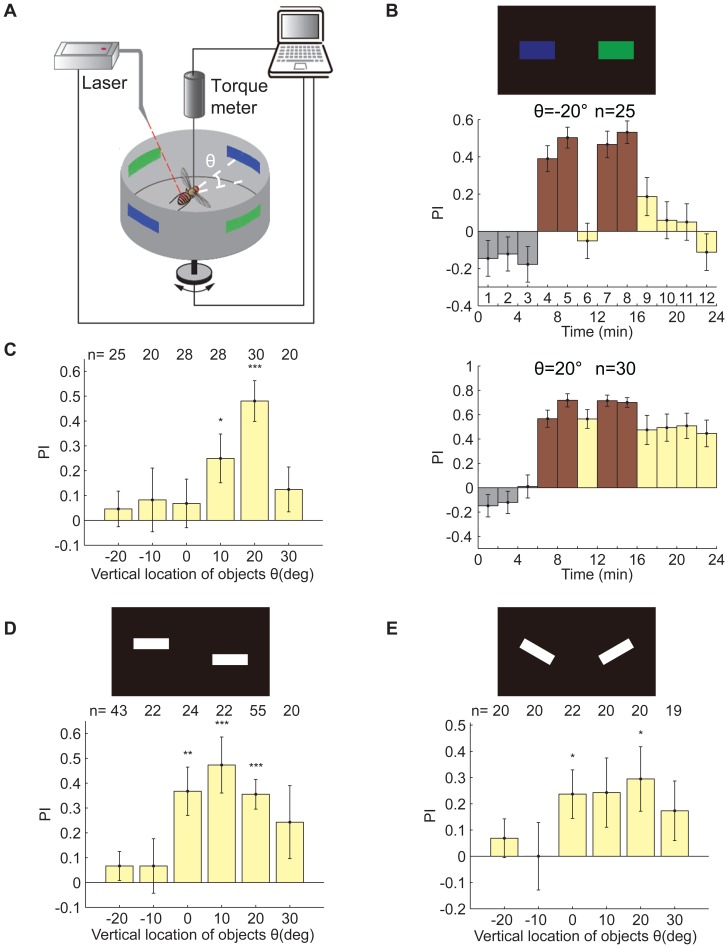
The effect of location on WTB flies' performance for memorizing different visual features. (A) Schematic of the flight simulator. Θ, elevation angle between the center of the patterns and the tethered fly. (B) Standard 24-min visual operant conditioning experiment to memorize a pair of colors (blue and green). Top panel, patterns used in this experiment. Middle panel, PIs of the flies when the patterns were 20° below them. The flies could not memorize which color was dangerous in this condition. Bottom panel, PIs of flies when the patterns were 20° above them. The flies could memorize which color was dangerous in this condition. (C) Test PIs when color bars were presented on different vertical locations. These PIs demonstrate an obvious location dependence (*p* = 0.0044). The acute zone for color memorization was 20° above the fly. (D) Top panel, the patterns used in these experiments, which consisted of bars on different vertical locations (ΔCOGs = 18°). Bottom panel, test PIs when these patterns were presented on different vertical locations. These PIs show significant location dependence (*p* = 0.0012). The acute zone for COGs memorization was 10° above the fly. (E) Top panel, the patterns used in these experiments, which consisted of bars of different contour orientation (ΔOrientation = 60°). Bottom panel, test PIs when these patterns were presented on different vertical locations. These PIs exhibit location dependence. However, this tendency was not significant (*p* = 0.4485). The acute zone for orientation memorization was 20° above the fly. The data are shown as the mean±SEM; the *p* values for location dependence were calculated by the Kruskal-Wallis test; significant differences between PIs and zero were calculated by the signed-rank test. * (*p*<0.05), ** (*p*<0.01) and *** (*p*<0.001).

The flies with hooks were fixed to a torque meter with a clamp in the center of the circular panorama (44 mm diameter) that could be rotated by a fast electric motor (Fig. 1A). The torque meter measured each fly's yaw torque around its vertical body axis and rotated the panorama around the fly by a negative feedback mechanism. This arrangement allowed the tethered flies to stabilize and choose their flight orientation with respect to the panorama by adjusting their yaw torque. The flies' yaw torque and the panorama's angular position (also referred to as the azimuth angle [20], the position of the object [21] and the angular distance [24]) were recorded continuously and stored in the computer (sampling rate, 20 Hz) for subsequent analysis. The panorama was divided into 4 quadrants, with the patterns at their respective centers. An infrared laser beam (10,600 nm) projecting to the abdomen of flies was switched on when flies oriented to the dangerous quadrants and was switched off when the flies oriented to the safe quadrants. Standard 24-min training paradigms consisted of 12 2-min blocks (Fig. 1B, middle panel): blocks 1–3, pre-training session; blocks 4, 5, 7 and 8, training session; blocks 6 and 9–12, test session. The patterns in this paradigm were on black or white backgrounds. Patterns in the opposite quadrants were identical; patterns in neighboring quadrants were different with respect to one visual feature (color, vertical position of center of gravity or orientation). The width of the color bars was 40°, and the height was 20°. The width of the black and white bars was 40°, and the height was 12°. These patterns were printed on ink jet transparency films with a color jet printer (Epson Stylus Photo 1390). The paper arena was illuminated by transmitted light from a white LED array. A TES 1330A digital lux meter was used to measure the illuminance. The illuminance of the white LED array was homogeneous in the horizontal direction, and varied from 540 lux to 480 lux in the down-up direction. The mean illuminance on the black background was 9 lux and the mean illuminance on the white background was 221 lux. In experiments using patterns on different vertical locations, different flies were trained.

The performance of tethered flies was evaluated quantitatively in 2-min bins using the “preference index” (PI), which was calculated as follows: PI = (t_1_–t_2_)/(t_1_+t_2_), where t_1_ is the time spent heading toward safe patterns and t_2_ is the time spent heading toward dangerous patterns. The ability of tethered flies to discriminate patterns was evaluated quantitatively by the discrimination values in the pre-training session (Fig. S2). If flies spontaneously prefer one pattern, there should be a 180° periodicity in the distribution of the panorama's angular position. We used fast Fourier analysis to determine the amplitude of the components in this distribution. The discrimination values were calculated as D = 2A_180_/(A_120_+A_72_) [22]. To directly evaluate the inclination of tethered flies to move away from dangerous patterns based on their yaw torques, the relative difference in the magnitude of the yaw torques (rDT_abs_) was calculated as rDT_abs_ = (T_abs_ D–T_abs_ S)/T_abs_W, where T_abs_ = ∑|T|/n_torques_, T is the value of yaw torque in arbitrary units, n_torques_ is the number of yaw torques generated in this area, D represents the dangerous area, S represents the safe area and W represents the whole panorama (Fig. S3). The frequency of spikes is the number of spikes generated at certain areas divided by the time spent in that area. The yaw torque data were low-pass filtered at 6 Hz before counting the torque spikes, and the spike threshold was set 3 s.d. away from 0. The relative difference in torque spike frequency (rDFS) was calculated as rDFS = (FS_D - FS_S)/FS_W, where FS is the frequency of spikes in a certain area and D, S and W represent the same areas as above (Fig. S4).

### Fixation paradigm

The flies with hooks were fixed to a torque meter by a clamp in the center of a circular panorama as described above. The angular position of the panorama and the yaw torque of the flies were recorded continuously and stored in the computer (sampling rate, 20 Hz) for subsequent analysis. There was only one stripe or bar in the whole panorama. The width and height of the stripes were 6° and 20°, respectively; the width and height of the bars were 40° and 12°, respectively. The stimuli were black stripes on white backgrounds, black bars on white backgrounds and white bars on black backgrounds. Patterns on different vertical locations were randomly presented to the same flies. The mean illuminances were identical to those for the operant conditioning paradigm.

The mean error distance (MED) [24], [30], [31] was used to quantify fixation performance, which is the average of the absolute value of angular position Ψ. The distribution of angular positions (from−180° to 180°) was divided into 72 intervals and calculated as the dwelling time. The tendency of the tethered flies to keep the object in the central visual field was evaluated by the rDT_abs_, which was calculated as rDT_abs_ = (T_abs_ P - T_abs_ C)/T_abs_W, where T_abs_ is defined as above, C represents the quadrant around the object, P represents the rest area and W represents the whole panorama. Similarly, the rDFS was calculated as rDFS = (FS_P - FS_C)/FS_W, where FS, C, P and W have the same definitions as above. On the black background, pseudo PI was calculated as pseudo PI = (t_1_ - t_2_)/(t_1_+t_2_), where t_1_ is the time spent in the quadrants around or opposite the object and t_2_ is the time spent in the other 2 quadrants.

### Optomotor response paradigm

The flies with hooks were clamped to a torque meter in front of an LCD screen (View Sonic VX2268wm, 120 Hz) on which the visual stimuli were presented. The distance between the LCD monitor and the fly was 95 mm. The stimulus was programmed with MATLAB and was generated at a rate of 120 frames per second. From center to periphery, the illuminance of the LCD monitor varied from 1.40 lux to 1.21 lux on a black background, from 272 to 261 on a white background, and from 80 to 70 on a gray background.

Visual stimuli were 110°×20° moving gratings with temporal frequency f = 2 Hz and spatial wavelength λ = 27.5° or a single 6°×20° moving stripe with velocity v = 55 °/s. Patterns on 9 vertical locations (−40°∼+40°) were presented to the same flies in a random order. The vertical location of a grating was defined by the elevation angle θ from the tethered flies to the center of the grating. Each visual stimulus lasted 20 s, during which the direction of moving gratings or a moving stripe changed every 2 s. Between each pair of stimuli, the flies were presented with a 10 s interval of noise distraction in which dots from the random-dot background jumped around erratically. Each dot consisted of 27×27 pixels and was equivalent to a visual angle of approximately 5° in the central visual field. All tested flies were naïve. Each stimulus was presented to each individual fly only once. Flies that paused during the tracking of a given stimulus were excluded from the results.

The yaw torques of the flies were recorded at a sampling rate of 120 Hz and stored on a computer for subsequent analysis. The torque amplitude was calculated as the difference between neighboring peak and valley values of yaw torques. The component of 0.25 Hz was calculated by fast Fourier transform from yaw torques.

### Statistical analyses

Normalized torque is shown as the mean±SD, PI and MED are shown as means±SEM, and the rest of the data are shown by box plot. We used the Kruskal-Wallis test to compare indices of fly performance in response to visual stimuli at different vertical locations. Rank-sum tests were used for comparison between 2 groups. Comparison between a group and zero was performed with the signed-rank test. Statistical analysis was performed using MATLAB. The sample size of each group is reported in the figure legends, and the significance levels of the post-hoc tests are shown in the figures. Asterisks indicate levels of significant differences (*p<0.05; **p<0.01; ***p<0.001).

## Results

### The acute zone for visual feature memorization against a black background

*Drosophila* can remember several pattern parameters [22], [32], and memory retrieval is independent of the retinal position of the patterns [33]; however, little is known about whether the position of patterns can affect memory acquisition. In this study, a visual operant conditioning paradigm in-flight simulator was used to test the ability of WTB flies to learn visual patterns at different vertical locations. Three visual features (pattern parameters) were selected: color, vertical locations of the centers of gravity (COGs) and contour orientation. Because *Drosophila* can learn to associate color with heat punishment only in the presence of a black background [23], these 3 features were all presented against a black background. Taking the visual feature color as an example, pairs of patterns (horizontal bars) with different colors (blue or green, Fig. 1B, top panel) were set 20° below the tethered flies (θ = −20°), and these bars were identical with respect to other features such as size, shape and vertical location. Tethered flies were presented with these bars distributed evenly in the 4 quadrants of the visual panorama in the flight simulator (Fig. 1A; see Materials and Methods). Flight dwelling in quadrants with bars of one color (e.g., blue) was coupled with laser-heat punishment, while the other color (green) was not. Fly performance was quantified by PI over 2-min bins and was calculated by dividing the time flies spent in the safe quadrants minus that in the dangerous (punished) quadrants by the total time. The flies gradually developed a robust avoidance of the bars associated with punishment during the training session (Fig. 1B, middle panel, blocks 4, 5, 7 and 8). However, these flies did not remember which color was dangerous after training because their test PIs were not significantly different from zero (Fig. 1B middle panel, blocks 9–12). Then, color bars were set 20° above the tethered flies (θ = 20°), and these tethered flies were able to remember to avoid the bars with the color that had been associated with laser punishment in the training session (Fig. 1B, bottom panel). These results suggest that the vertical location of patterns does affect memory acquisition in the operant conditioning paradigm.

To study the location effect more precisely, we changed the vertical location of the color bars from −20° to +30° by 10° steps. The mean PIs of the test session for these locations are shown in Fig. 1C. There was a significant difference among these PIs (*p* = 0.0044, Kruskal-Wallis test), indicating that tethered flies could only learn to associate heat punishment with colors in a narrow area of their upper visual field. Next, we checked another visual feature, COGs. As shown in the top panel of Fig. 1D, ΔCOGs of 2 neighboring bars was 18°, and the vertical location of this set of patterns was defined by the average of the vertical locations of 2 neighboring bars in the panorama. When the vertical location of this set of patterns was changed from −20° to +30°, step by step, a significant change was also observed among the mean test PIs of tethered flies trained with this set of patterns (*p* = 0.0012, Kruskal-Wallis test, Fig. 1D, bottom panel). The tendency of these mean test PIs was similar to that observed with color bars; however, the former reached a peak at θ = 10° (Fig. 1D, bottom panel), while the latter had a maximum value at θ = 20° (Fig. 1C, bottom panel). Finally, we trained flies to associate laser punishment with the third visual feature, contour orientation. Pairs of bars with different contour orientations (the angle between the horizontal plane and one orientation was +30°, while that of the other orientation was −30°, yielding ΔOrientation = 60°, Fig. 1E, top panel) were presented to tethered flies at the 6 vertical locations mentioned above. The mean test PIs for these locations also exhibited tendencies similar to that described for the visual feature COGs, with a maximum value at θ = 20°, although there was no significant location dependence (*p* = 0.4485, Kruskal-Wallis test; Fig. 1E, bottom panel). In summary, these results suggest that there is an acute zone in the upper visual field (10–20° above the equator of the compound eyes) of WTB flies that is necessary for memorizing the visual features of patterns on a black background.

### The learning effect indicated by the yaw torque magnitude in the training session

**Figure 2:**
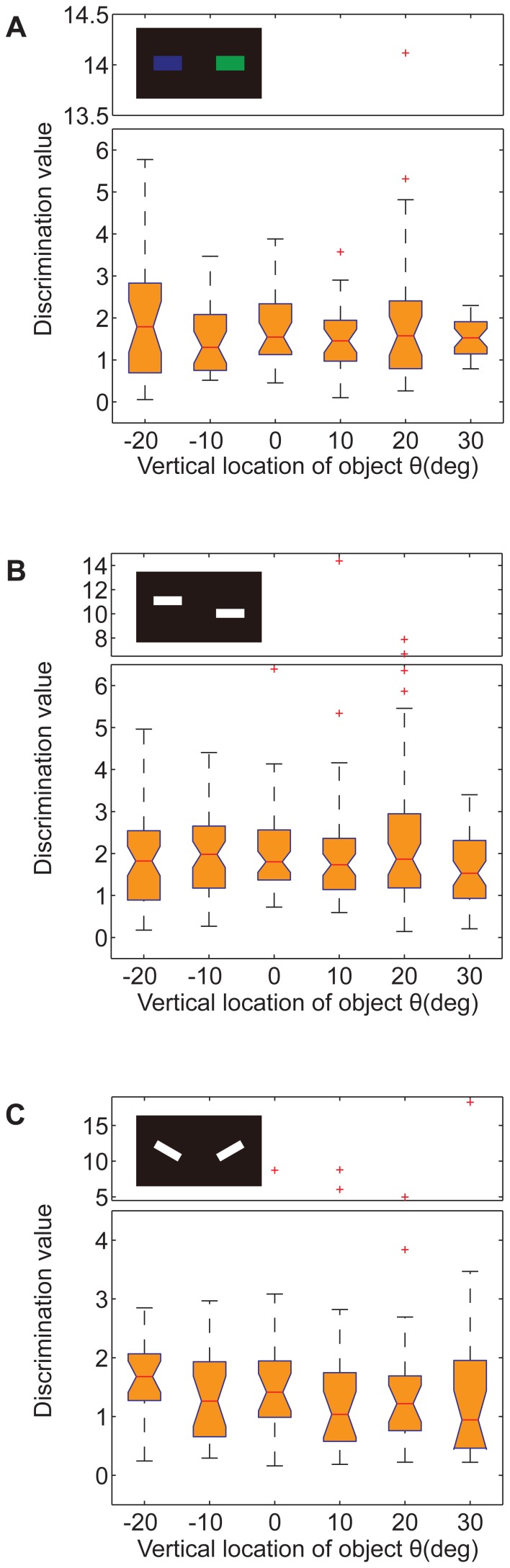
The ability of tethered flies to discriminate patterns was unaffected by the location of the patterns. There was little location dependence among the discrimination values. (A) Box plots of discrimination value for color, *p* = 0.8771. (B) Box plots of discrimination value for COG, *p* = 0.5929. (C) Box plots of discrimination value for contour orientation, *p* = 0.4949. Patterns are presented as inserts. The data are shown by box plot; the *p* values were calculated by the Kruskal-Wallis test; the red crosses represent outliers; the y-axes of the charts are truncated for compactness.

To better understand the role of the acute zone in the upper visual field of WTB flies, the results described above were analyzed in detail. Because the vertical location might affect the test performance of the flies by affecting their ability to discriminate patterns, we analyzed the discrimination value in the period before training (pre-training session) (Fig. S2). For all these 3 visual features, there was neither a significant location dependence nor a tendency similar to that of the mean test PIs in the flies' ability to discriminate patterns (Fig. 2); the *p* values were 0.8771, 0.5929 and 0.4949 for color, COGs and contour orientation, respectively (Kruskal-Wallis test). Thus, the acute zone for visual feature memorization did not result from variations in discrimination ability at different vertical locations.

**Figure 3:**
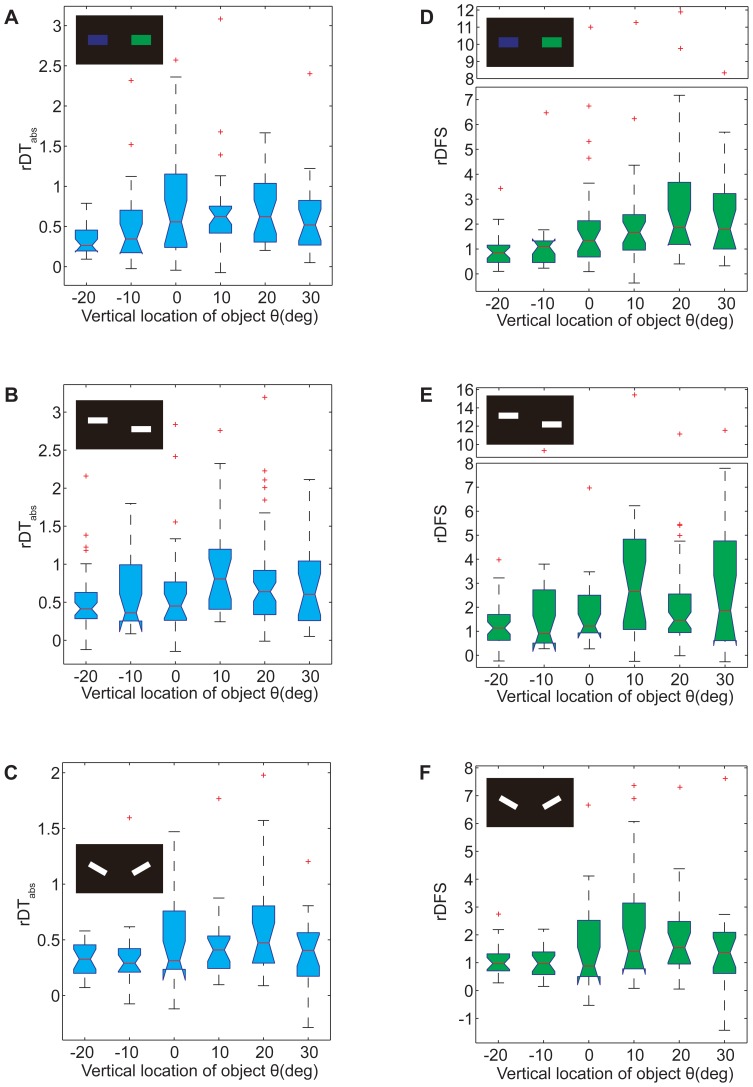
Location effect represented by torque analysis in the visual operant paradigm. (A–C) Obvious location dependence in the rDT_abs_ between safe quadrants and dangerous quadrants. (A) Box plots of rDT_abs_ for color, *p* = 0.0022. (B) Box plots of rDT_abs_ for COG, *p*  = 0.0189. (C) Box plots of rDT_abs_ for contour orientation, *p* = 0.2243. (D–F) Significant location dependence in the rDFS between safe quadrants and dangerous quadrants. (J) Box plots of rDFS for color, *p* = 4.9328×10^−4^. (K) Box plots of rDFS for COG, *p* = 0.0023. (L) Box plots of rDFS for contour orientation, *p* = 0.0257. Patterns are presented as inserts. The data are shown by box plot; the *p* values were calculated by the Kruskal-Wallis test; the red crosses represent outliers; the y-axes of certain charts are truncated for compactness.

The yaw torques of the tethered flies represented the rawest data collected from the flight simulator, and it has been reported that wild-type Canton-S flies modulate the magnitude of yaw torques in conditioning [34]. Therefore, we also analyzed the yaw torques of the tethered flies in the training session. The rDT_abs_ value was used to quantify the preference for the safe pattern over the dangerous pattern based on yaw torques (Fig. S3). Using the visual feature color as an example, the median of rDT_abs_ first ascended then descended from the vertical location −20° to +30°, with a maximum value at the location θ = 10° (Fig. 3A). The change tendency of the rDT_abs_ median was similar to that of the mean test PIs (Fig. 1C), which had a larger value for the positions above tethered flies (θ>0°) than that at the same height or below tethered flies (θ ≤ 0°), although they reached peak values at different locations. There was a significant difference in the distribution of rDT_abs_ at different locations (*p* = 0.0022, Kruskal-Wallis test). For the visual features COGs and contour orientation, the change tendency of the rDT_abs_ median was also similar to that of the mean test PIs (Fig. 2B&C and Fig. 1D&E), and they had maximum values at the same location. There was a significant difference in the distribution of rDT_abs_ at different locations for the visual feature COGs (*p* = 0.0189, Kruskal-Wallis test) but not for contour orientation (*p* = 0.2243, Kruskal-Wallis test), just as for the mean test PIs. These results suggest that rDT_abs_ in the training session somehow indicated the learning effect of tethered flies for patterns at these vertical locations.

Like saccades in free flying, the flies simultaneously generated torque spikes (short pulses of large torque) that rotated the panorama with large angles in the flight simulator (Fig. S4A). Therefore, the frequency of torque spikes may play an important role in the mean size of the yaw torque. We used rDFS value to quantify this effect (Fig. S4). In fact, the change in the rDFS median had the same tendency as the mean test PIs (Fig. 3D-F), with maximum values at the same locations. The distribution of rDFS varied significantly at these locations, from −20° to +30°, even for the visual feature contour orientation (*p* = 0.0257; *p* = 4.9328×10^−4^ for color and *p* = 0.0023 for COGs, Kruskal-Wallis test). This result indicates that rDFS is more sensitive to the location of patterns than test PIs, so we assumed that the frequency of torque spikes excited by laser punishment was an important index of the learning effect and that it could be affected by pattern location.

### The effect of the elevation angle on visual feature memorization

**Figure 4:**
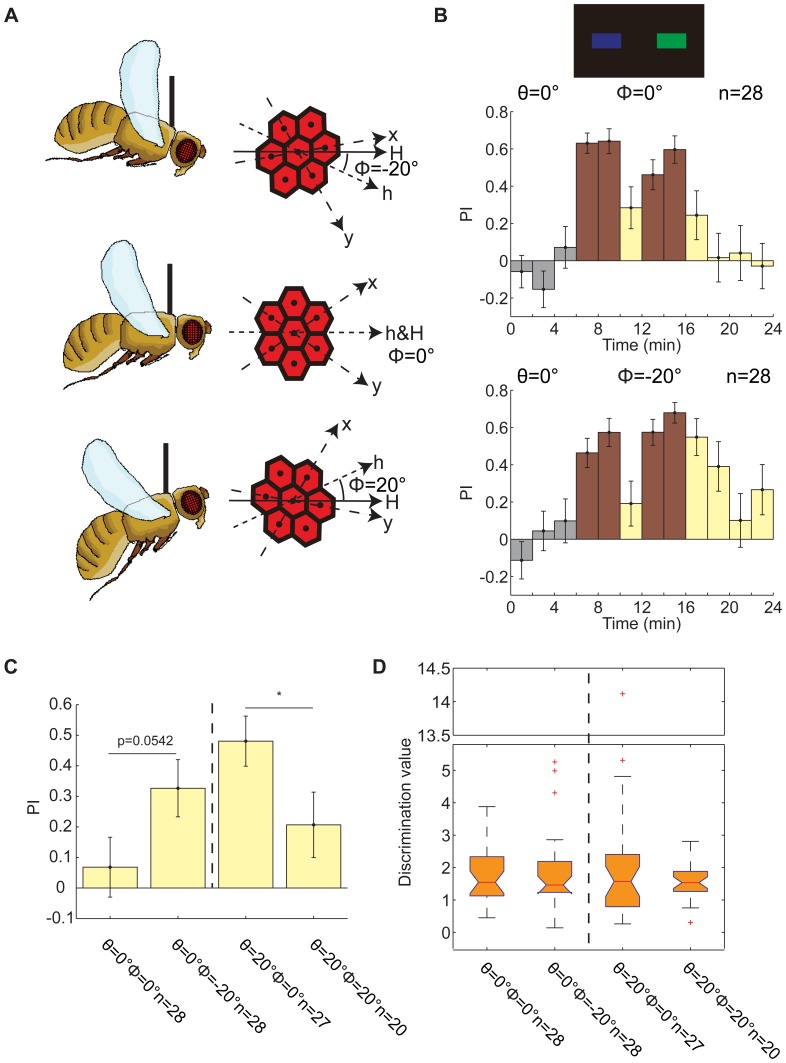
Learning effects can be altered by the elevation angles of tethered flies' heads. (A) Schematic of tethered flies and their compound eyes with 3 elevation angles. h, the latitudinal axis of the ommateum; Φ, the elevation angle between h and the horizontal plane H. (B) Standard color memorization experiment in which patterns were presented on the vertical location Θ = 0°. Top panel, patterns used in this experiment. Middle panel, PIs of flies when the elevation angle Φ was 0°. The flies could not memorize which color was dangerous in this condition. Bottom panel, PIs of flies when Φ = −20°. Flies could memorize which color was dangerous in this condition. (C) The test PIs could be affected by the elevation angle at both locations (Θ = 0°; Θ = 20°). (D) The elevation angle had little influence on the flies' ability to discriminate colors. *p* = 0.9152 for the location θ = 0°; *p* = 0.8976 for the location θ = 20°. The y-axis of this chart is truncated for compactness. The data in (B&C) are given as the mean±SEM; the data in (D) are shown by box plot; the red crosses represent outliers; significant differences were calculated by the rank-sum test; * (*p*<0.05).

Because visual stimuli from different positions project to different ommatidia [17], we assumed that the ability of tethered flies to learn patterns depends, at least in part, on the position of the ommatidia to which these patterns projected. To test this assumption, we modulated the elevation angle Φ of the heads of the tethered flies. The elevation angle was defined by angle between the equator of compound eyes and the horizontal plane (Fig. 4A). When the elevation angle is lowered, the pattern will project to more superior ommatidia. Tethered flies were trained to associate color with laser punishment at 2 vertical locations, at the same height as or 20° above the flies. At the former location, the test PIs of the flies were not significantly different from zero (*p* = 0.5847, signed-rank test) (Fig. 4B, middle panel) when the equator of their eyes was on the horizontal plane (Fig. 4A, middle panel). However, if the heads were lowered by 20° (Fig. 4A, top panel), the color bar directly in front of the flies projected to the ommatidia 20° above the equator of their eyes. Thus, the flies learned which color meant danger in this case (*p* = 0.0038, signed-rank test; Fig. 4B, bottom panel), just as the flies with a normal elevation angle did when the color bars were 20° above them (Fig. 1B, bottom panel). At the latter location, the flies' heads were raised by 20° so that the color bar was projected to the ommatidia around the equator of their eyes when it was at the 12 o'clock position for the tethered flies. Flies could not learn to discriminate the dangerous color in this condition: their test PIs were not significantly different from zero (*p* = 0.0674, signed-rank test). In summary, the tethered flies' test performances could be improved by decreasing the elevation angle of their heads at θ = 0°, although the improvement was not significant (*p* = 0.0542, rank-sum test; Fig. 4C, left part), and significantly impaired by increasing the elevation angle at the location θ = 20° (*p* = 0.0424, rank-sum test; Fig. 4C, right part). We also determined whether the elevation angle of the heads could affect the ability of flies to discriminate colors; however, there was no significant change in the discrimination values at either the location θ = 0° (*p* = 0.9152, rank-sum test; Fig. 4D, left part) or the location θ = 20° (*p* = 0.8976, rank-sum test; Fig. 4D, right part). These results indicate that the change in pattern location can be mimicked by modulating the elevation angle of the flies' heads, suggesting that the acute zone for visual feature memorization is composed of certain ommatidia in the upper part of the compound eye.

### The candidate acute zone for visual feature memorization against a white background

**Figure 5:**
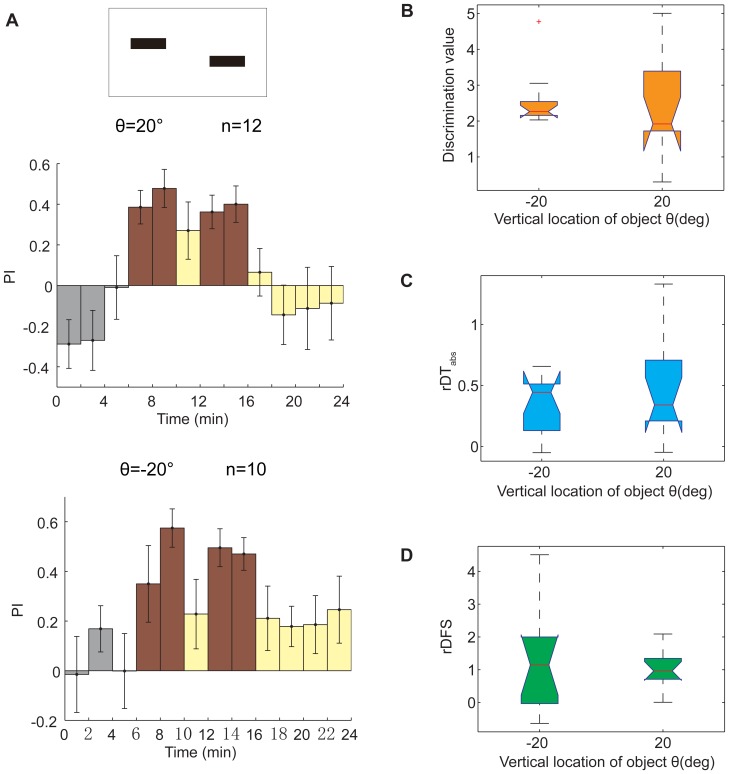
A lower location was preferred for COGs memorization against a white background. (A) A standard 24 min visual operant conditioning experiment to memorize COGs with a white background. Top panel, patterns used in this experiment (ΔCOGs = 20°). Middle panel, PIs of flies when the patterns were 20° above them. The test PIs of the flies were not different from zero (*p* = 0.5186) in this condition. Bottom panel, PIs of flies when the patterns were 20° below them. Flies showed a tendency to avoid dangerous patterns in the test session for this condition. The test PIs at the location θ = −20° were higher than those at the location θ = +20°, although the difference was not significant (*p* = 0.0927). These PIs were not significantly different from zero (*p* = 0.084). (B–D) There was no significant difference in the other 3 indices between the 2 locations. (B) Discrimination value, *p* = 0.1563. (C) The rDT_abs_ between safe quadrants and dangerous quadrants, *p* = 0.7667. (D) The rDFS between safe quadrants and dangerous quadrants, *p* = 0.8691. The data in (A) are given as the mean±SEM; the data in (B–D) are shown by box plot; the red cross represents outlier; significant differences between PIs and zero were judged by the signed-rank test; significant differences between 2 groups were judged by the rank-sum test.

We also examined the location effect on a white background. We trained flies to learn COGs at 2 vertical locations (θ = +20° & −20°), and the results suggested that flies memorize visual features in their lower visual field better with a white background than with a black background (Fig. 5A). The mean test PI was higher at θ = −20° (PI = 0.2052) than at θ = +20° (PI = −0.0699), although the difference was not significant (*p* = 0.0927, rank-sum test). Neither PI was significantly different from zero (*p* = 0.0840 for θ = −20° and *p* = 0.5186 for θ = +20°, sign-rank test). There were no significant differences in the distributions of the discrimination value, the rDT_abs_ or the rDFS at these 2 locations (Fig. 5B-D, *p* = 0.1563 for the discrimination value, *p* = 0.7667 for the rDT_abs_ and *p* = 0.8691 for the rDFS, rank-sum test), in contrast to the results with the black background. When we trained other flies using different locations, especially θ = 0°, we found that flies had a strong preference to the low bars. If the low bars were coupled with laser punishment, the flies persisted in trying to return to the dangerous quadrants. It appeared that their preference could not be reversed by training. However, if the high bars were coupled with laser punishment, the flies rarely went to the dangerous quadrants during the entire experiment. These data suggested that the training was invalid at these locations. Therefore, these data were not considered for further analyses. Further studies are required to understand the different effects of black and white backgrounds.

### The relationship between the background and preferred vertical location for object fixation behavior

**Figure 6:**
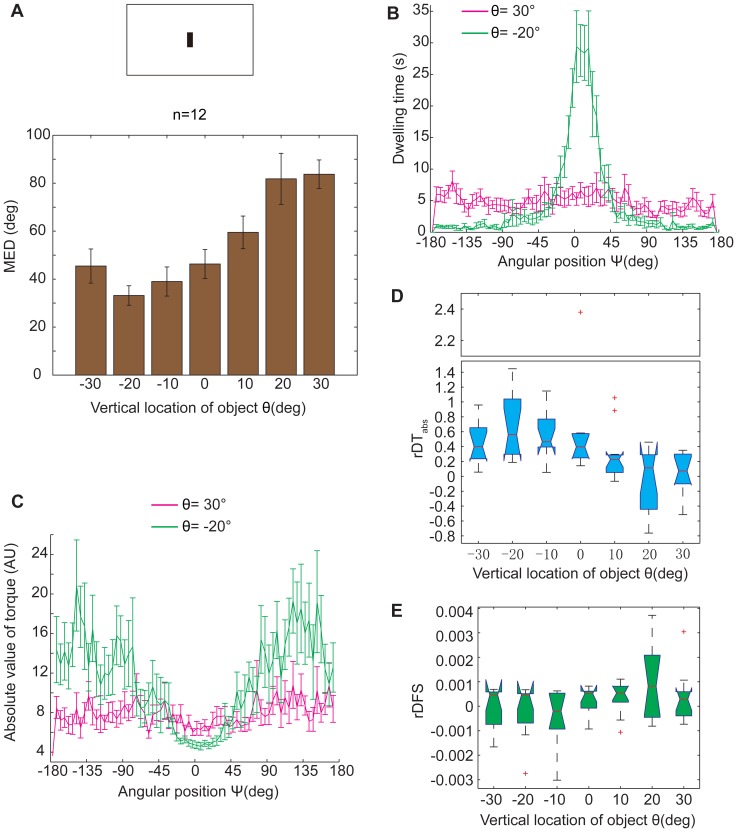
The location effect of a black stripe on the fixation performance of WTB flies. (A) The MED, which was a contrarian indicator of fixation performance, had a significant location dependence (*p* = 4.0868×10^−6^). The tethered flies showed the best fixation performance when the small black stripe was 20° below them. (B&C) The fixation performance at 2 locations (−20°; 30°) was selected to show more detail. (B) The distribution of the dwelling time in the whole panorama in a 6 min experiment. The distribution of dwelling time had an obvious peak in the central quadrant (−45∼+45° area around the stripe in the panorama) on the location θ = −20°; the distribution of dwelling time was quite even at the location θ = 30°. (C) The distribution of the mean of yaw torque magnitude in the whole panorama. Yaw torque magnitude decreased in the central quadrant and increased in the rest of the area at the location θ = −20°; yaw torque magnitude was more or less the same in the whole panorama at the location θ = 30°. (D) Obvious location dependence in the rDT_abs_ between the central quadrant and the rest area (*p* = 2.0731×10^−4^). The chart has a truncated y axis for compactness. (E) There was no obvious location dependence in the rDFS between the central quadrant and the rest area (*p* = 0.1699). These differences were quite small compared to those of torque magnitude. The data in (A–C) are given as the mean±SEM; the data in (D&E) are shown by box plot; the red crosses represent outliers; the *p* values were calculated by the Kruskal-Wallis test.

In the flight simulator, flies spontaneously orient themselves to a black stripe in a phenomenon known as “object fixation behavior” [17], [24], [25]. In the visual operant conditioning paradigm, flies also orient to certain patterns spontaneously or after training. It is therefore likely that some relationship exists between visual feature memorization and visual object fixation. When the vertical location of a small black stripe (W = 6°, H = 20°) on a white background was changed from −30° to +30°, WTB flies modulated their inclination to keep the stripe in front of them. This inclination was quantified by MED (see Materials and Methods). A significant difference was observed in the distribution of MED values at these locations (*p* = 4.0868×10^−6^, Kruskal-Wallis test), and the mean MED reached its lowest value at θ = −20° (Fig. 6A), indicating that flies fixed on the black stripe best at this location. Two locations, −20° and +30°, were selected to gain greater insight into fixation behavior. A black stripe at the former location could attract flies to orient to it, and there was an obvious peak in the flies' dwelling time between the angular position −45° and +45° (Fig. 6B, green curve). However, the flies did not exhibit a clear fixation to the black stripe at θ = +30°, and their dwelling time was approximately the same in any position (Fig. 6B, purple curve). We also analyzed the yaw torques at these 2 locations and observed that tethered flies modulated their torque magnitude according to their angular position only at the former location (Fig. 6C, green curve). At the latter location, similar to the dwelling time, the flies' yaw torque magnitude was approximately the same in any position (Fig. 6C, purple curve). Considering the comparison of the distribution of dwelling time and torque magnitude at these 2 locations (Fig. 6B&C), rDT_abs_ and rDFS could be calculated as above (Fig. S3 & S4), by comparing the yaw torques in the quadrants around the visual object with the yaw torques in the rest area. We analyzed the rDT_abs_ on these locations from −30° to +30°. A significant difference was also observed in the distribution of rDT_abs_ (*p* = 2.0731×10^−4^, Kruskal-Wallis test), and this distribution exhibited a tendency that was the opposite of what was observed for the MED (Fig. 6D). Thus, the rDT_abs_ was also a good index with which to quantify tethered flies' fixation inclination. Interestingly, the result of rDFS analysis was different from that of the rDT_abs_ analysis. The values of rDFS were 3 orders of magnitude lower than those of rDT_abs_ (Fig. 6E), and there was no significant difference in their distribution at different vertical locations (*p* = 0.1699, Kruskal-Wallis test). Thus, unlike the training session in the visual operant conditioning paradigm, tethered flies' torque spike generation was not affected by the angular position or the vertical location of the visual object in the object fixation paradigm.

**Figure 7:**
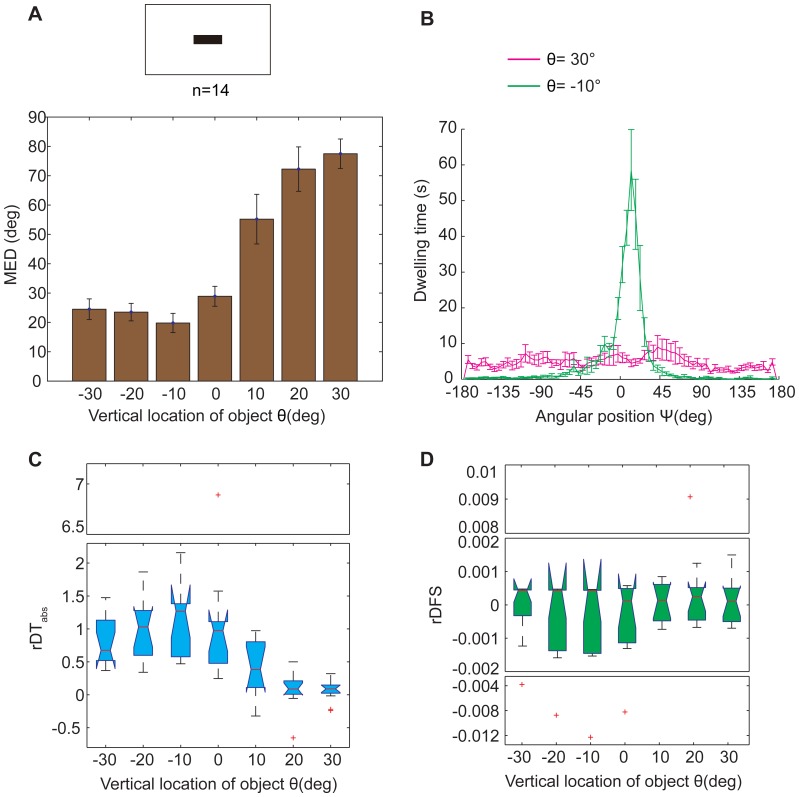
The location effect of a black bar on the fixation performance of WTB flies. (A) The MED had a significant location dependence (*p* = 1.4509×10^−9^). The best location for black bar fixation was 10° below the flies. (B) The fixation performance at 2 locations (−10°; 30°) was selected to show more details of the dwelling time in the whole panorama. Note the sharp peak in the dwelling time in the central quadrant (−45∼+45° area around the bar in the panorama) at the location θ = −10°; the distribution of dwelling time was quite even at the location θ = 30°. (C) Obvious location dependence in the rDT_abs_ between the central quadrant and the rest area (*p* = 1.1145×10^−8^). (D) There was no obvious location dependence in the rDFS between the central quadrant and the rest area (*p* = 0.7273). These differences were quite small. The data in (A&B) are given as the mean±SEM; the data in (C&D) are shown by box plot; the red crosses represent outliers; the *p* values were calculated by the Kruskal-Wallis test; the y-axes of certain charts are truncated for compactness.

To make the object fixation paradigm more similar to the visual operant conditioning paradigm, we changed the vertical stripe to a horizontal bar of the same size as the white bar in the visual operant conditioning paradigm (Fig. 7A). More significant differences were observed among the fixation indices for flies fixed to the bar at these locations from −30° to +30° (*p* = 1.4509×10^−9^ for MED and *p* = 1.1145×10^−8^ for rDT_abs_, Kruskal-Wallis test), and these indices were better at most locations than those for flies fixed to the stripe (Fig. 7A&C and Fig. 6A&D), although the best location for object fixation shifted from −20° to −10°. Taking the best location as an example, the distribution of dwelling time had a steeper peak when flies fixated on the bar than when flies fixated to the stripe (Fig. 7B and Fig. 6B, green curve). The frequency of torque spikes did not depend on either the angular position or the vertical location of the visual object (Fig. 7D, *p* = 0.7273, Kruskal-Wallis test). These results indicated that tethered flies fixed on the bar better than on the stripe on the white background. It is noteworthy that there was a large difference between the MEDs at θ = +10° and −10°. Considering that the high bar was located at θ = +10° and the low bar was located at θ  =  −10° in the COGs memory experiment on a white background, these results explain why flies had such a strong preference to the low bar at θ  =  0°.

**Figure 8:**
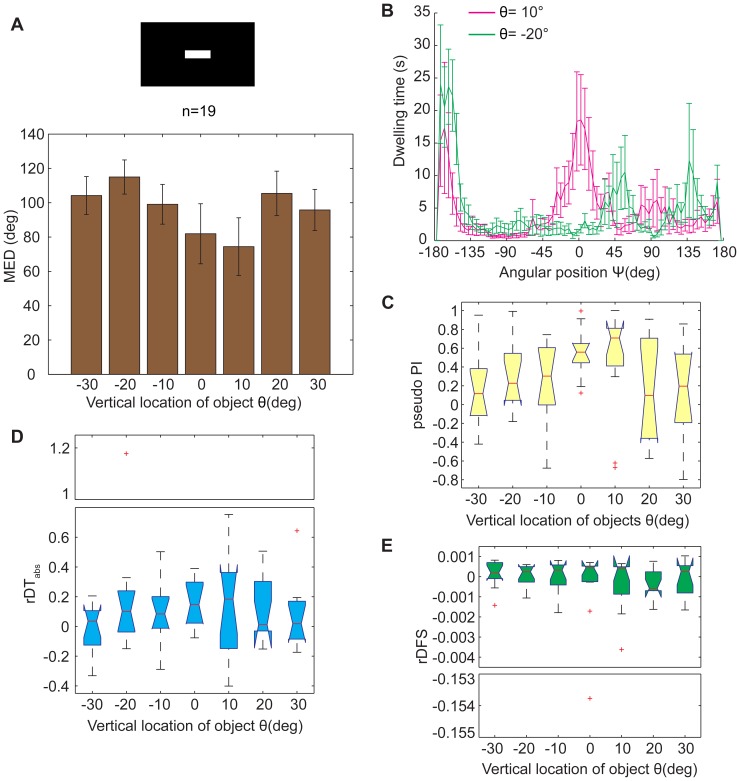
The location effect was reversed with a black background. (A) The MED had an insignificant location dependence (*p* = 0.6259). The preferred location for white bar fixation was 10° above the flies. (B) The fixation performance at 2 locations (−20°; 30°) was selected to show additional details of the dwelling time in the whole panorama. There were 3 peaks in the distribution of the dwelling time (around −180°, +45° and +135° to the bar, respectively) at the location θ = −20°; there were 2 peaks in the distribution of the dwelling time (around and opposite the bar, respectively) at the location θ = 10°. (C) Box plots of pseudo PI. There was some location dependence in the pseudo PIs, although this tendency was not significant (*p* = 0.1044). (D) There was an insignificant location dependence in the rDT_abs_ between quadrants around or opposite the bar and the rest area (*p* = 0.5959). (E) There was no obvious location dependence in the rDFS between quadrants around or opposite the bar and the rest area (*p* = 0.6124). These differences were quite small. The data in (A&B) are given as the mean±SEM; the data in (C–E) are shown by box plot; the red crosses represent outliers; the *p* values were calculated by the Kruskal-Wallis test; the y-axes of certain charts are truncated for compactness.

It has been reported that flies avoid white stripes on black backgrounds and that they tend to turn their backs to such stripes [17]. Because we had trained the flies to discriminate white bars at different vertical locations against a black background, we also assessed the flies' anti-fixation performance with white bars on a black background (Fig. 8A, *p* = 0.6259, Kruskal-Wallis test). A white bar was repulsive to flies at most locations (mean MED>90°), and the MED reached its lowest point at the location θ = 10°. This result suggested some correlation with the learning paradigm (Fig. 1D). Two locations, −20° and +10°, were selected to provide greater insight into this anti-fixation behavior. At the location θ = −20°, the dwelling time had an obvious peak at Ψ of approximately −180°, indicating the inclination of the flies to avoid the white bar. However, there were also small peaks in dwelling time at the angular positions Ψ = 45° and Ψ = 135°, and thus, the flies did not simply avoid the bar but had complex responses to the white bar against the black background. It should be emphasized that the dwelling time exhibited peaks at both the positions Ψ = −180° and Ψ = 0° on the vertical location θ = 10°, indicating that a white bar could be both attractive and repulsive at this location. Considering that identical patterns were located in these 2 positions in the visual operant conditioning paradigm, we assumed that the quarters around the positions Ψ = −180° and Ψ = 0° were “safe quarters” and that the other 2 quarters were “dangerous quarters,” allowing us to calculate pseudo PI here similar to PI in the visual operant conditioning paradigm (Fig. 8C, *p* = 0.1044, Kruskal-Wallis test). The changing tendency of pseudo PI was similar to that of PI in the visual operant conditioning paradigm for most locations, with the exception of θ = 20° (Fig. 8C and Fig. 1D). We could also calculate rDT_abs_ and rDFS here like PI in the visual operant conditioning paradigm. The changing tendency of rDT_abs_ here was also similar to that in the visual operant conditioning paradigm (Fig. 8D and Fig. 3B), although there was no significant difference in the distribution of rDT_abs_ at different locations (*p* = 0.5959, Kruskal-Wallis test). The distribution of rDFS here was similar to that when flies fixed to a black stripe or horizontal bar on a white background (Fig. 8E, *p* = 0.6124, Kruskal-Wallis test). These results explain, at least partially, why the test PI reached a peak value at the location θ = 10° in Fig. 1D.

### The effect of the background illuminance on optomotor response

**Figure 9:**
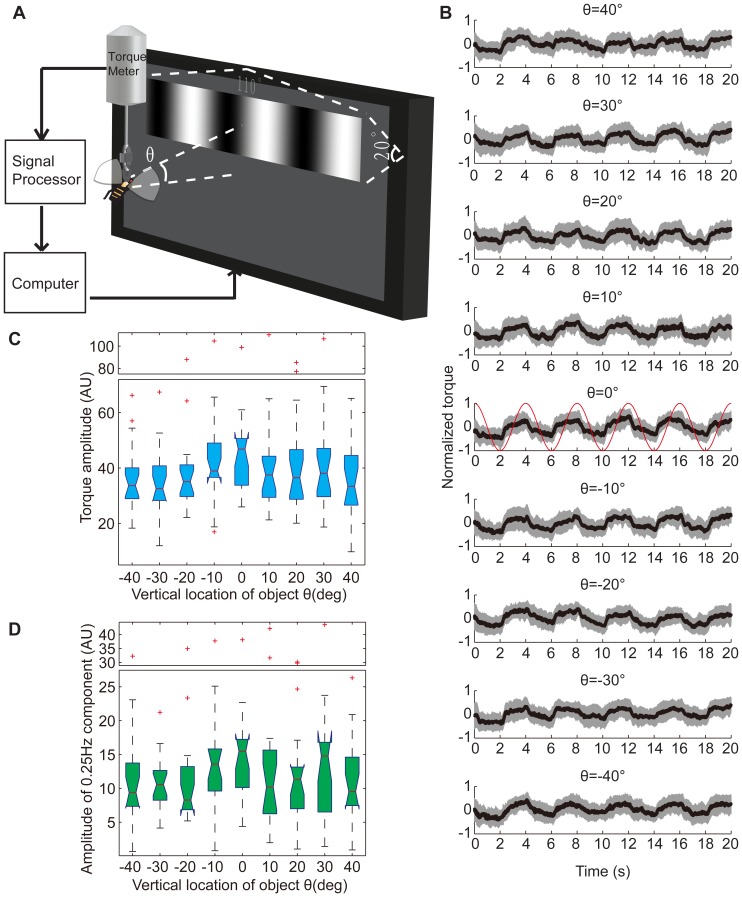
Effects of grating location on the optomotor responses of WTB flies. (A) Schematic of the experimental setup for testing the optomotor response of *Drosophila*. Θ, elevation angle between the center of the grating and the tethered fly. (B) Periodic yaw torque responses to gratings at different vertical locations. Yaw torques were normalized. The black curve is the mean of the yaw torques (n = 19); the gray shadow is the standard deviation of the yaw torques. From top to bottom, the vertical location Θ of the gratings changed from +40° to −40°; the red curve in the middle panel was the sine curve used as the ideal response model. (C&D) The acute zone for optomotor response is near the equator of the visual field. (C) Box plots of the amplitudes of the yaw torques at all 9 vertical locations. There was no significant location dependence (*p* = 0.3887). (D) Box plots of the amplitudes of the 0.25 Hz component of the yaw torques, which were calculated by fast-Fourier transform. There was no significant location dependence (*p* = 0.3418). The *p* values were calculated by the Kruskal-Wallis test; the red crosses represent outliers; the y-axes of certain charts are truncated for compactness.

Although the *Drosophila* eye does not contain obvious large facets, its ommatidia are not homogeneously distributed. Because the local efficiency of an elementary motion detector (EMD) is related to the distribution of ommatidia [17], the acute zone for visual feature memorization may originate from an acute zone for motion detection. However, it has been reported that the densest area of ommatidia is in neither the upper nor the lower portion of the compound eye but in the frontal area, near the equator, of the compound eye [17]. To elucidate the relationship between visual feature memorization and motion detection, the optomotor responses of WTB flies were evaluated using sine gratings moving left or right on a gray background at different vertical locations, using the experimental arrangement shown in Fig. 9A. The yaw torques of flies were recorded and normalized. The tethered flies periodically changed their yaw torque direction following the gratings' moving direction at all vertical locations (from +40° to −40°, Fig. 9B and Fig. S5A&C). A sine curve was used as an ideal response model (Fig. 9B, red curve in the middle panel), and its peaks and valleys can be followed well by the normalized yaw torque. The amplitude of the flies' yaw torque was calculated to quantitatively evaluate the flies' optomotor performance (Fig. 9C). No significant difference was observed between the amplitudes of the yaw torques at these vertical locations (*p* = 0.3887, Kruskal-Wallis test). Because the cycle of the visual stimuli was 4 s, a fast Fourier transform was used to obtain the 0.25 Hz component of the yaw torques (Fig. 9D). No significant difference was observed between their amplitudes (*p* = 0.3418, Kruskal-Wallis test). The median amplitude had a maximum value at θ = 0° for both yaw torques themselves and their 0.25 Hz component, a distribution that is identical to that reported for ommatidia. The tendency of the distribution of torque amplitudes was somewhat symmetrical around θ = 0°. However, the distribution of 0.25 Hz component amplitudes was not symmetrical and exhibited another peak around θ = 30°.

**Figure 10:**
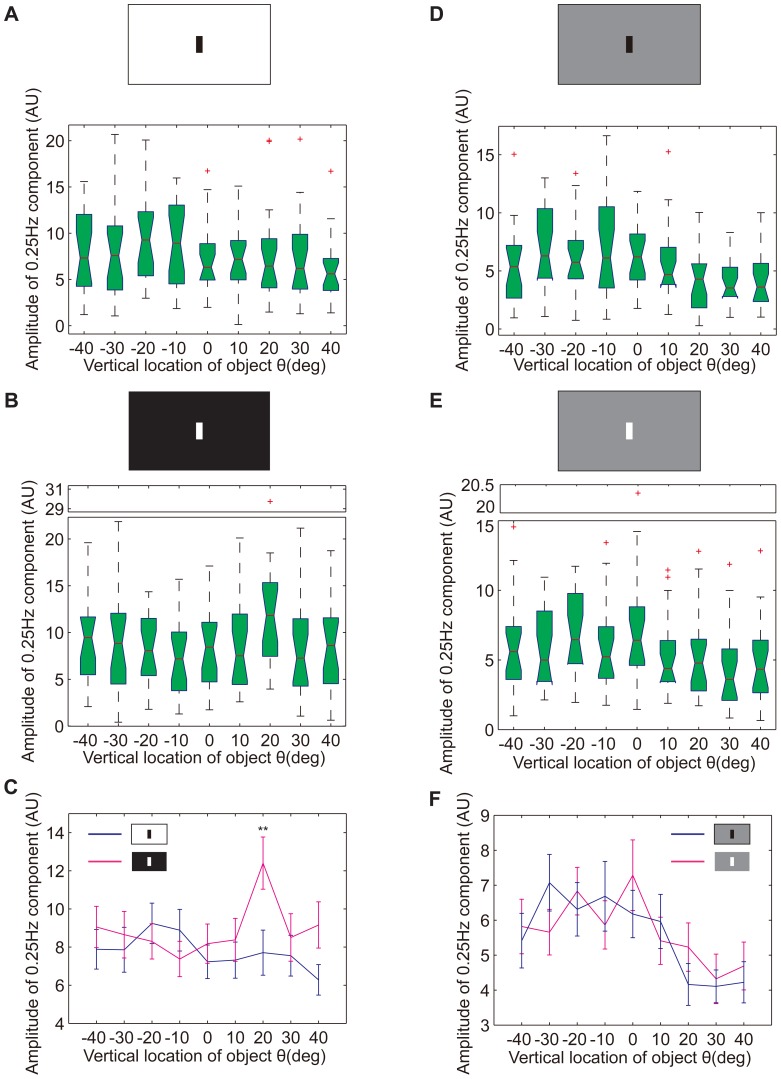
Effects of background illuminance and panorama contrast on the optomotor responses of WTB flies. (A) Box plots of the amplitudes of the 0.25 Hz component of the yaw torques recorded from the optomotor response following a single black stripe on a white background (n = 20). The preferred location was in the lower visual field. There was no significant location dependence (*p* = 0.6653). (B) Box plots of the amplitudes of the 0.25 Hz component of the yaw torques recorded from the optomotor response following single white stripe on a black background (n = 20). The preferred location was in the upper visual field. There was no significant location dependence (*p* = 0.2625). (C) Data in (A&B) are shown as the mean±SEM. There was significant difference at θ = 20° (*p* = 0.0066). Blue line, black stripe on a white background; purple line, white stripe on a black background. (D) Box plots of the amplitudes of the 0.25 Hz component of the yaw torques recorded from the optomotor response following single black stripe on a gray background (n = 20). The preferred location was in the lower visual field. There was a significant location dependence (*p* = 0.0177). (E) Box plots of the amplitudes of the 0.25 Hz component of the yaw torques recorded from the optomotor response following single white stripe on a gray background (n = 20). The preferred location was also in the lower visual field. There was no significant location dependence (*p* = 0.0798). (F) Data in (D&E) are shown as the mean±SEM. Their distributions were similar for all 9 locations. Blue line, black stripe on a gray background; purple line, white stripe on a gray background. The *p* values for location dependence were calculated by the Kruskal-Wallis test; significant difference between two groups was judged by the rank-sum test; the red crosses represent outliers; the-y axes of certain charts are truncated for compactness; ** (*p*<0.01).

The location effect on optomotor response to gratings was not similar to those that were observed in the visual operant conditioning or visual object fixation paradigms. This inconsistency may be due to the gray background or the difference between the sine gratings and the stripe or bars. To eliminate these interferences, a black stripe moving left or right on a white background was used as visual stimuli. However, the tethered flies did not follow the stripe as well as the gratings (Fig. S5). It was difficult to calculate the torque amplitude in certain cases. Therefore, only the 0.25 Hz component amplitude was used to quantify the optomotor response. The median of this amplitude reached a maximum value at θ = −20° (Fig. 10A), similar to the MED in the visual object fixation paradigm (Fig. 6). However, there was no significant difference between the locations (*p* = 0.6653, Kruskal-Wallis test). We then reversed the contrast between the stripe and the background. Just as in the former 2 paradigms, the preferred location shifted to the upper visual field at θ = 20° (Fig. 10B). Although there was no significant difference in the 0.25 Hz component amplitudes between the locations (*p* = 0.2625, Kruskal-Wallis test), the amplitudes were significantly higher on a black background than that on a white background at θ = 20° (*p* = 0.0066, rank-sum test; Fig. 10C).To obtain a better understanding of the effect of contrast reversal, we examined the optomotor response of tethered flies to a black or white stripe moving on a gray background. The contrast was somewhat reversed in these 2 conditions. A significant difference between the 9 examined locations was observed with the black stripe (*p* = 0.0177, Kruskal-Wallis test; Fig. 10D) but not with the white stripe (*p* = 0.0798, Kruskal-Wallis test; Fig. 10E). Surprisingly, the distribution of the 0.25 Hz component amplitudes was generally the same in these 2 conditions (Fig. 10D-F). Both of the distributions had their maximum values in the lower visual field. These results suggest that the background illuminance plays a more important role than contrast in affecting the preferred location.

## Discussion

In this study, we used a flight simulator to investigate the ability of WTB flies to memorize visual features at different vertical locations. Visual features could be memorized by tethered flies only when the patterns with those features were presented in a narrow area of the upper visual field (Fig. 1C-E). However, the ability of flies to discriminate these features did not depend on the vertical location of the patterns (Fig. 2). Memory retrieval in *Drosophila* has been reported to be independent of the retinal position of the pattern being recalled [33]. We conclude, therefore, that a special vertical location of patterns is required for memory acquisition of visual features by WTB flies. In fact, tethered flies modulated their torque magnitude or torque spike frequency according to whether the laser punishment was on or off in the training session, and this process was dependent on the vertical location of the patterns (Fig. 3). This phenomenon is unlikely to correlate with the distribution of ommatidia because the densest area of ommatidia is around the equator of the compound eye [17]. With a white background, flies spontaneously orient to a black stripe [17], [24], [25]; however, the fixation behavior we observed indicated a preference for the stripe or bar below the flies (Fig. 6 and Fig. 7), as has been reported for the house fly [21]. Because the flies also memorized patterns below them better than patterns above them (Fig. 5), this result suggests that visual feature memorization and visual object fixation have the same optimal locations. This assumption was also supported by the results with a black background. The response of flies to a white stripe or bar on a black background is complex and can be both attractive and repulsive (Fig. 8B) [17], and the best location for white bar fixation is also the best location for memorization of the visual feature COGs (Fig. 8A and Fig. 1D). Similar results were also observed for the optomotor response. The best location was in the lower visual field on a white background and in the upper visual field on a black background (Fig. 10A-C).These results suggest an innate correlation among visual feature memorization, visual object fixation and optomotor response in the location effect.

The best vertical locations for memorization of these 3 visual features were not identical (Fig. 1C-E), possibly because distinct pathways are involved in the processing of different visual features, as there are distinct memory traces for different visual features [22]. However, a simpler explanation is that this inconformity is derived from the vertical span of the patterns. The vertical span of patterns used in color or orientation memorization is 20°. However, for the visual feature COGs, the vertical span is more complex: the vertical span is 30° when all 4 patterns in the panorama are considered, while that of each single pattern is 12°. When flies were trained to memorize the visual feature orientation, their performance did not exhibit any significant location variation tendency. Because the patterns used in the orientation memorization had no vertical contour, they were relatively difficult for the flies to fixate upon, impairing the learning effect. In fact, the best performance of orientation memorization was lower than that of color or COGs memorization, and thus the incline of the contour might be responsible for the indistinct location effect. By modulating the elevation angle of the flies' heads, the change in the patterns' vertical location could only be mimicked, not replaced (Fig. 4C). A possible explanation for this result is that the elevation angle could only obviously affect the visual stimuli directly ahead, leaving the visual stimuli in the lateral visual field largely unaffected. This is particularly likely for the visual stimuli in the angular position Ψ = 90°, which projected to certain ommatidia regardless of the elevation angle.

Fixation behavior can be modulated by attention [24]. It has been reported that the attention effect could be observed for cues in the lower visual field even when the cues precede the test stimulus by several seconds and is spatially separated from the test stimulus in the presence of a bright background [20]. It therefore appears that the lower visual field more easily draws flies' attention on the bright background. The experiments in the previous study were performed with a green LED arena and most of our experiments were performed using a paper arena that was illuminated by transmitted light from a white LED array. However, it is very likely that visual objects in the lower visual field could also attract more attention from flies within our experimental arrangement on a white background. Therefore, the spatial distribution of attention may be involved in the location effect of object fixation, at least on a bright background. However, more work is required to test this hypothesis. Inverting the contrast of the panorama can make flies change and even invert their yaw torques [17]. These phenomena may explain why flies prefer visual objects in the upper visual field against a black background.

In the optomotor response experiment, we observed that flies exhibited a maximum yaw torque response to gratings at θ = 0° (Fig. 9). In addition to the distribution of ommatidia and EMDs, there may be another neural basis for this phenomenon. For example, the horizontal system neurons in the lobula plate, which are involved in optomotor response, share the receptive field around the equator [35]. However, the flies could not follow a single moving stripe as well as moving gratings (Fig. S5), a possible explanation for this result is that single stripe covered fewer EMDs than did the gratings. The optomotor response to a moving stripe could be reduced by panorama contrast decrease, although the preferred location was nearly unaffected by panorama contrast inversion (Fig. 10). In *Drosophila*, L1 and L2 neurons are involved in the ON and OFF edge motion detection, respectively [36]. Therefore, the decrease in the optomotor response that occurs due to contrast lowering may reflect the efficiency of L1 and L2 neurons in different contrast conditions [26]. Our results suggest that the preferred location for optomotor response is primarily affected by background illuminance. However, despite the use of the same background, the optomotor responses while following moving gratings or a single moving stripe were not identical with respect to their preferred location (Fig. 9D and Fig. 10D&E). Without the visual object, the background illuminance reached a peak in the central visual field in the optomotor paradigm, and had a maximum value in the lower visual field in the visual operant conditioning paradigm and visual object fixation paradigm. Considering the moving visual object, the distribution of the panorama illuminance was too complex to be analyzed. Therefore, more studies are required to clarify how the contrast and panorama illuminance affect the response of tethered flies to visual stimuli in certain locations.

The 3 paradigms we used here are also widely used to study visual processing mechanisms of flies [26] as well as mechanisms of advanced cognitive behavior [9], [10], [24], [27], [28]; however, little of the literature concerns the locations of visual stimuli. We systematically analyzed the location effect for the first time, and our results indicate that small variations in the location of visual stimuli or in flies' elevation angle might lead to significant changes in the behavior performance of flies. Therefore, strict control of the retinal position of visual stimuli should be standard henceforth in studies using these paradigms, so that the results will be more robust and consistent. The comparison of the location dependence of behavior performances provided some insight into the correlation among these paradigms, about which little was previously known. One effect of training in the flight simulator is the change of inclination to orient to certain patterns. It is thus not surprising that there was some correlation between the location effects of patterns in the visual feature memorization task and the visual object fixation task. These results about preferred locations predict some overlap in the visual information processing pathways for these 3 behaviors. A hypothesis for the overlap is the retinotopic distribution of attention. Visual stimuli might draw more attention when they projected to certain ommatidia. Therefore, flies might perform better when facing visual stimuli at certain location. However, it is likely that the patterns attract attention more easily after the training in the visual operant conditioning paradigm, and that the distribution of attention becomes more homogenous due to the ceiling effect. Therefore, only memory acquisition, but not memory retrieval, is retinotopic. Despite the correlation mentioned above, our results also suggest that different pathways for processing and transferring visual information underlie these behaviors. The analysis of the torque spikes (saccades) suggested that the torque spike modulation was particularly necessary for memory acquisition of visual features. Spontaneous saccades represent an active search strategy for odor tracking in free flight [37], and saccades triggered by visual expansion are necessary for collision avoidance. Therefore, it is not unreasonable that saccades are involved in avoiding dangerous patterns and searching for safe patterns. Distinct visual features are stored in different areas of the central complex [22], which also controls locomotion in *Drosophila* [38], making the central complex an appropriate candidate for the control of the generation of saccades in response to visual stimuli at different locations.

## Supporting Information

Figure S1 **How to adjust the head elevation angle of a tethered fly.** Top panel, a tethered fly with a clamp was fixed to the electrode holder in an MF-830 microforge. The handle of the clamp was in vertical orientation. The elevation angle of the fly head was measured by a scale eyepiece in the MF-830 microforge. Bottom panel, the elevation angle of the fly head was confirmed to be zero after the angle between the hook and the clamp was adjusted appropriately.
(TIF)

Figure S2 **The definition of discrimination value.** (A) A sample of the distribution of dwelling time on different angular positions in the pre-training session. (B) The amplitudes of the components with different periods as calculated by fast Fourier analysis. (C) The discrimination value was calculated by doubling the amplitude of the 2-cycle component of the angular position distribution and dividing this value by the sum of the amplitudes of the 3-cycle and 5-cycle components.
(TIF)

Figure S3 **The definition of rDT_abs_.** (A) An example of 30 seconds yaw torque trace of a tethered fly in the training session. Red line, yaw torque in the dangerous quadrants; green line, yaw torque in the safe quadrants. (B) The average of torque absolute value (T_abs_) in the training session. Red bar, T_abs_ in the dangerous quadrants (T_abs_D); green bar, T_abs_ in the safe quadrants (T_abs_S); blue bar, T_abs_ in the whole panorama (T_abs_W). (C) The relative difference in the T_abs_ (rDT_abs_) was calculated using the following formula: T_abs_D minus T_abs_S, divided by T_abs_W.
(TIF)

Figure S4 **The definition of rDFS.** (A) An example of 15 seconds yaw torque trace with torque spikes in the training session. Red line, the spike threshold which was set 3 s.d. away from 0. In this example, three torque peaks and one torque valley beyond the red line were counted as torque spikes. The torque valley which had a value of nearly -100 was an obvious torque spike. (B) The frequency of torque spikes (FS) in the training session. Red bar, FS in the dangerous quadrants (FS_D); green bar, FS in the safe quadrants (FS_S); blue bar, FS in the whole panorama (FS_W). (C) The relative difference in the FS (rDFS) was calculated using the following formula: FS_D minus FS_S, divided by FS_W.
(TIF)

Figure S5 **The comparison between gratings and single stripe in optomotor responses.** (A) An example of periodic yaw torque response to gratings at θ = 0°. (B) The statistical result of the periodic yaw torque responses to gratings at θ = 0° (n = 19). The black curve is the mean of the yaw torques; the gray shadow is the standard error of the yaw torques. (C) An example of yaw torque response to a single moving stripe at θ = 0°. The periodicity is less clear. (D) The statistical result of the yaw torque responses to a single moving stripe at θ = 0° (n = 20). The black curve is the mean of the yaw torques; the gray shadow is the standard error of the yaw torques.
(TIF)
