# Supplementary figures and images for: Distinct Acute Zones for Visual Stimuli in Different Visual Tasks in Drosophila
Source: PLoS One. 2013 Apr 9;8(4):e61313. doi: 10.1371/journal.pone.0061313 (PMC3621824; doi:10.1371/journal.pone.0061313)

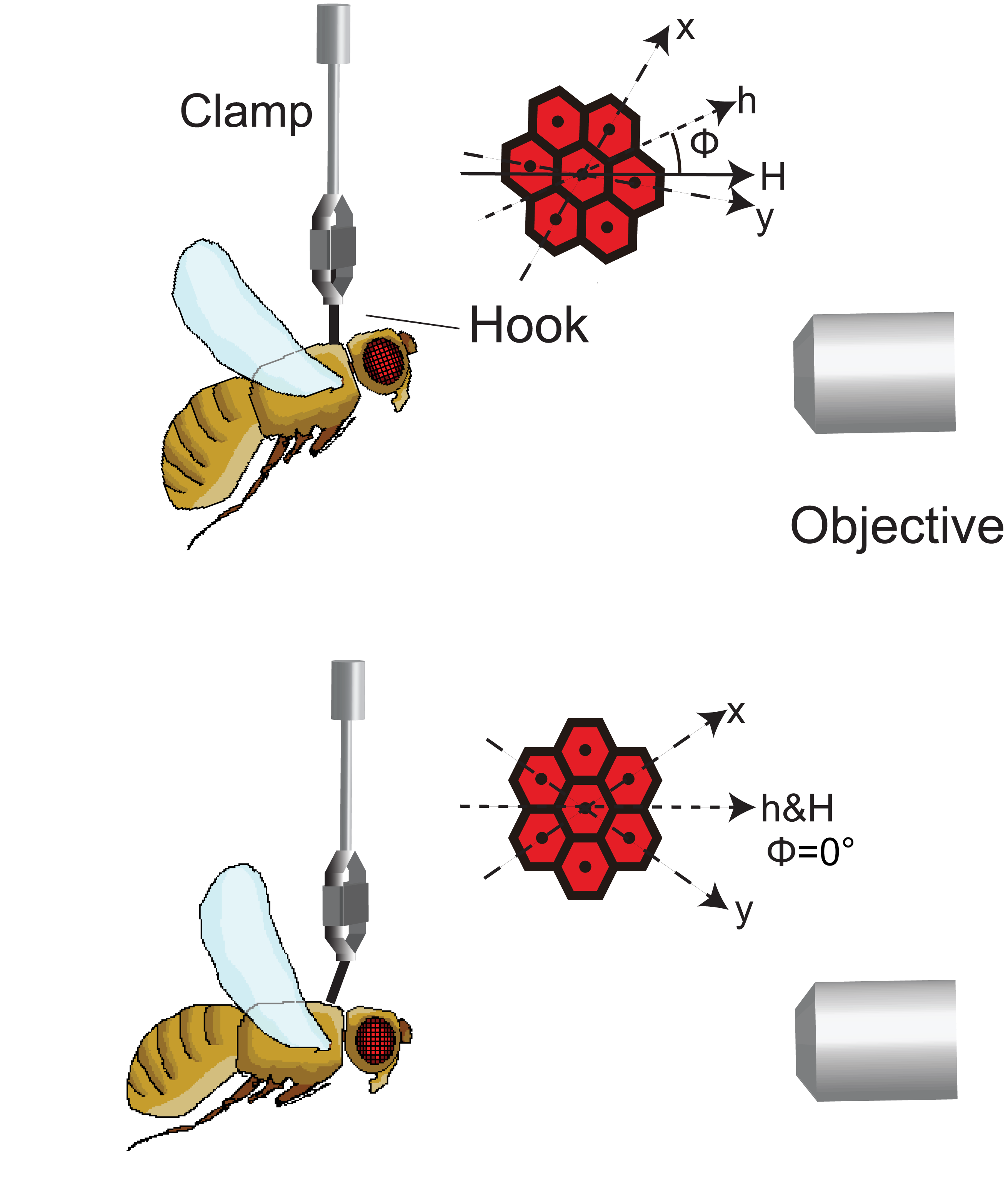

Supplement: Figure S1 — How to adjust the head elevation angle of a tethered fly. Top panel, a tethered fly with a clamp was fixed to the electrode holder in an MF-830 microforge. The handle of the clamp was in vertical orientation. The elevation angle of the fly head was measured by a scale eyepiece in the MF-830 microforge. Bottom panel, the elevation angle of the fly head was confirmed to be zero after the angle between the hook and the clamp was adjusted appropriately. (TIF) [file pone.0061313.s001.tif]

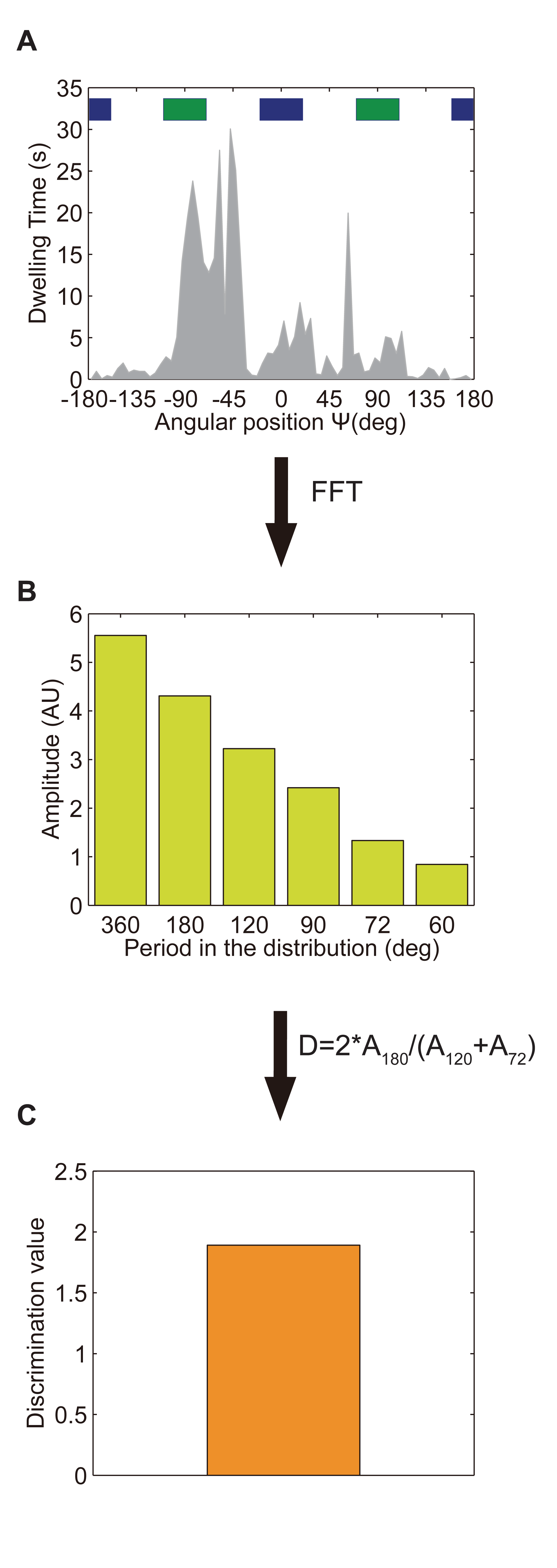

Supplement: Figure S2 — The definition of discrimination value. (A) A sample of the distribution of dwelling time on different angular positions in the pre-training session. (B) The amplitudes of the components with different periods as calculated by fast Fourier analysis. (C) The discrimination value was calculated by doubling the amplitude of the 2-cycle component of the angular position distribution and dividing this value by the sum of the amplitudes of the 3-cycle and 5-cycle components. (TIF) [file pone.0061313.s002.tif]

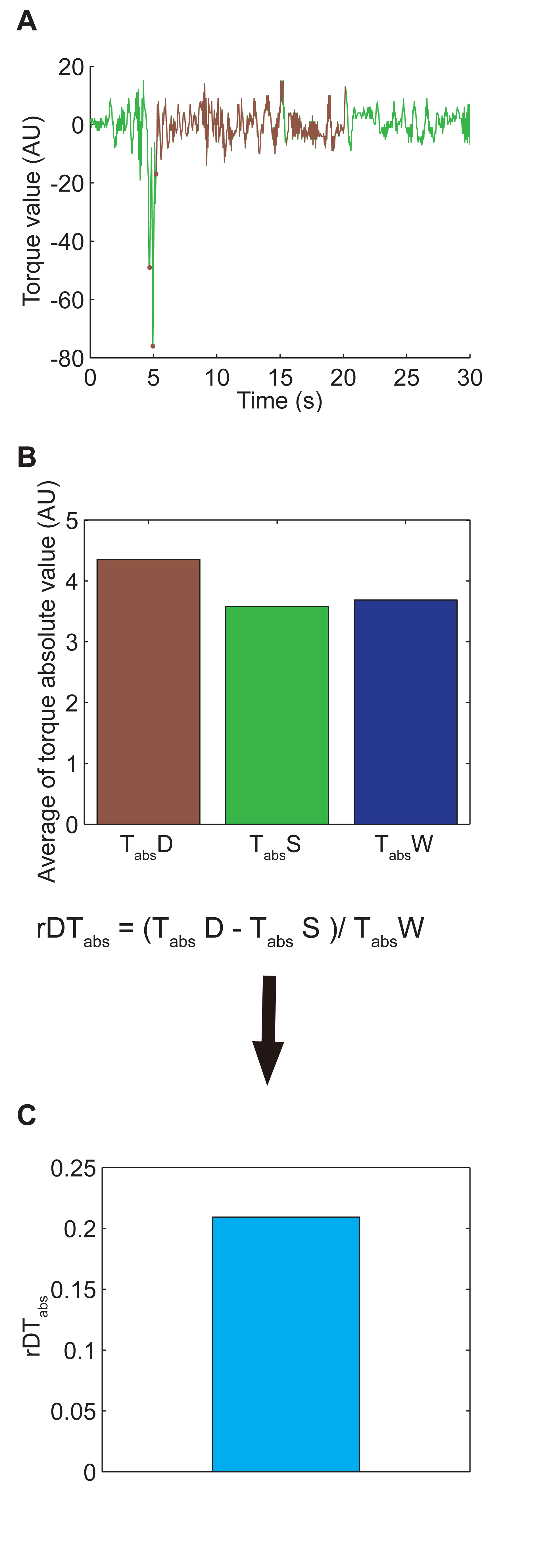

Supplement: Figure S3 — The definition of rDTabs. (A) An example of 30 seconds yaw torque trace of a tethered fly in the training session. Red line, yaw torque in the dangerous quadrants; green line, yaw torque in the safe quadrants. (B) The average of torque absolute value (Tabs) in the training session. Red bar, Tabs in the dangerous quadrants (TabsD); green bar, Tabs in the safe quadrants (TabsS); blue bar, Tabs in the whole panorama (TabsW). (C) The relative difference in the Tabs (rDTabs) was calculated using the following formula: TabsD minus TabsS, divided by TabsW. (TIF) [file pone.0061313.s003.tif]

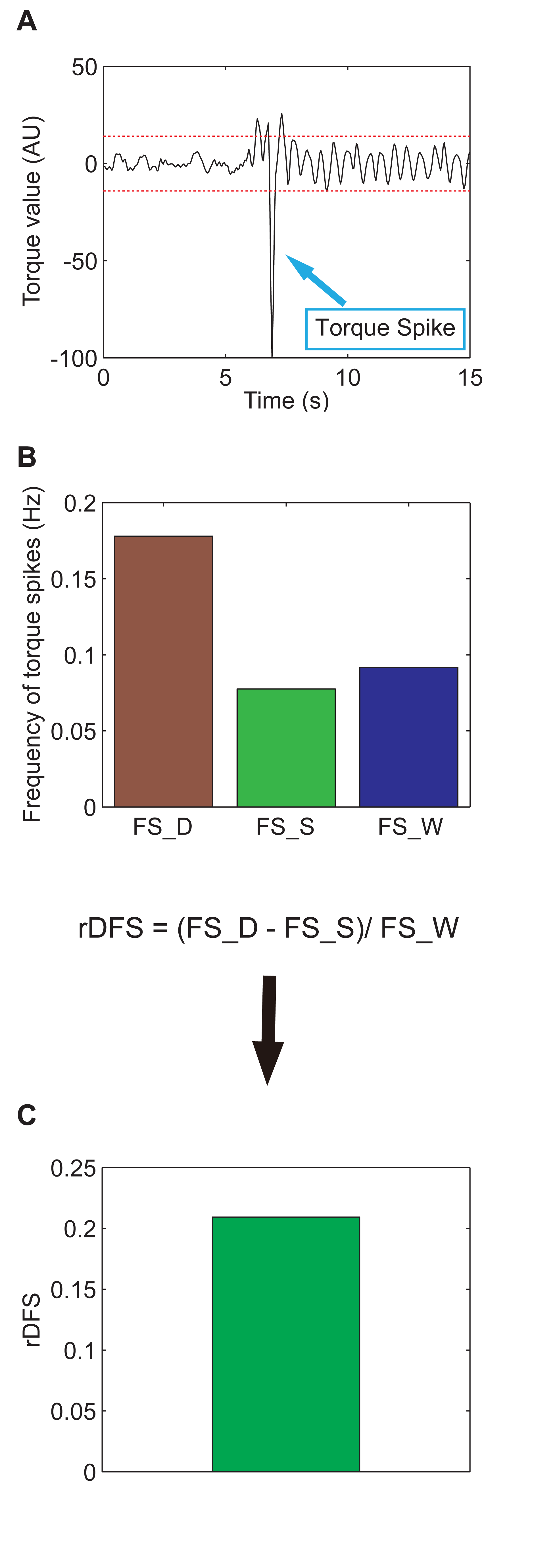

Supplement: Figure S4 — The definition of rDFS. (A) An example of 15 seconds yaw torque trace with torque spikes in the training session. Red line, the spike threshold which was set 3 s.d. away from 0. In this example, three torque peaks and one torque valley beyond the red line were counted as torque spikes. The torque valley which had a value of nearly -100 was an obvious torque spike. (B) The frequency of torque spikes (FS) in the training session. Red bar, FS in the dangerous quadrants (FS_D); green bar, FS in the safe quadrants (FS_S); blue bar, FS in the whole panorama (FS_W). (C) The relative difference in the FS (rDFS) was calculated using the following formula: FS_D minus FS_S, divided by FS_W. (TIF) [file pone.0061313.s004.tif]

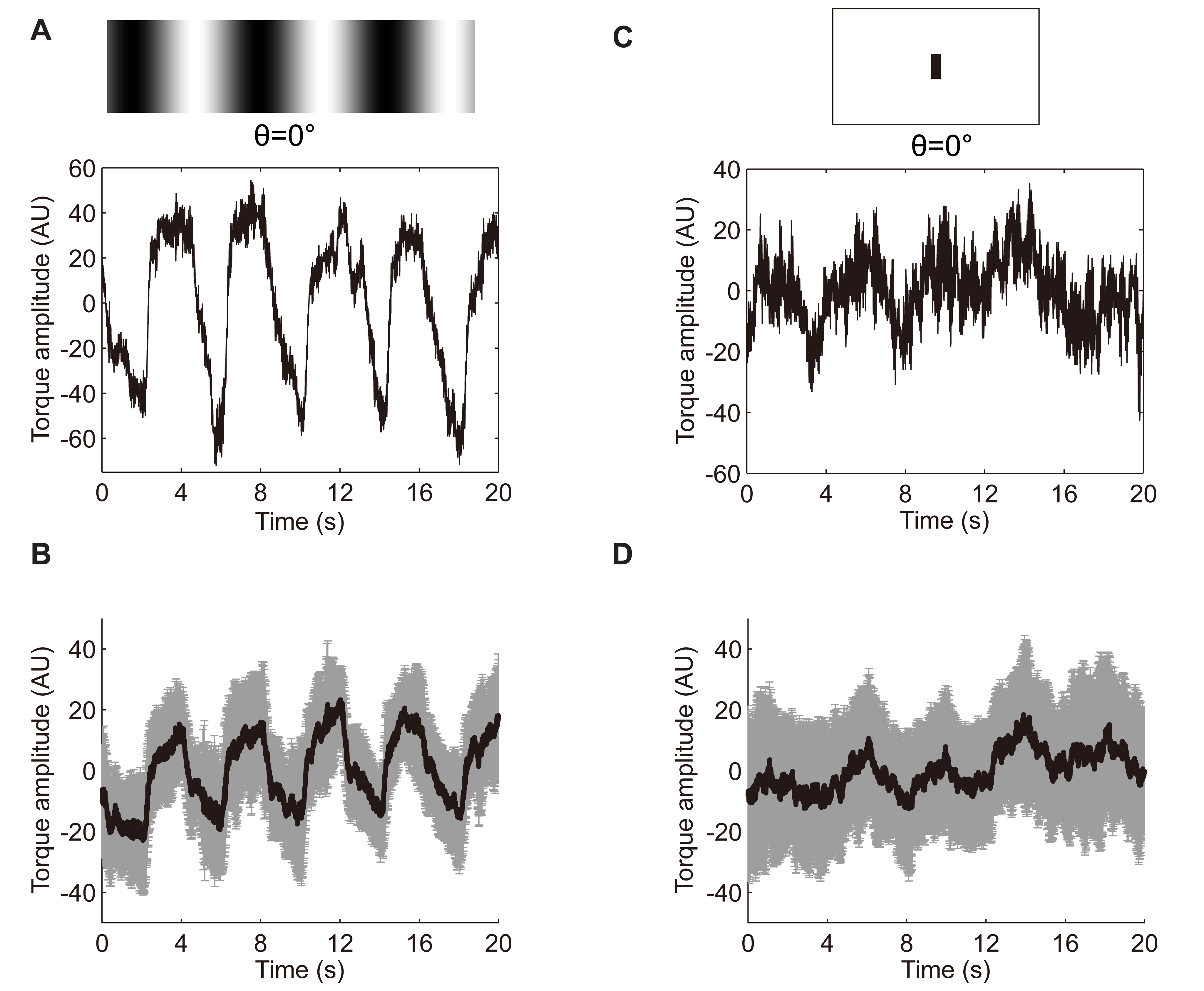

Supplement: Figure S5 — The comparison between gratings and single stripe in optomotor responses. (A) An example of periodic yaw torque response to gratings at θ = 0°. (B) The statistical result of the periodic yaw torque responses to gratings at θ = 0° (n = 19). The black curve is the mean of the yaw torques; the gray shadow is the standard error of the yaw torques. (C) An example of yaw torque response to a single moving stripe at θ = 0°. The periodicity is less clear. (D) The statistical result of the yaw torque responses to a single moving stripe at θ = 0° (n = 20). The black curve is the mean of the yaw torques; the gray shadow is the standard error of the yaw torques. (TIF) [file pone.0061313.s005.tif]
